# Valorization of Lignocellulosic Wastes to Produce Phytase and Cellulolytic Enzymes from a Thermophilic Fungus, *Thermoascus aurantiacus* SL16W, under Semi-Solid State Fermentation

**DOI:** 10.3390/jof7040286

**Published:** 2021-04-09

**Authors:** Keerati Tanruean, Watsana Penkhrue, Jaturong Kumla, Nakarin Suwannarach, Saisamorn Lumyong

**Affiliations:** 1 Biology Program, Faculty of Science and Technology, Pibulsongkram Rajabhat University, Phitsanulok 65000, Thailand; keerati.t@psru.ac.th; 2 School of Preclinical Science, Institute of Science, Suranaree University of Technology, Nakhon Ratchasima 30000, Thailand; watsanapenkhrue@gmail.com; 3 Department of Biology, Faculty of Science, Chiang Mai University, Chiang Mai 50200, Thailand; Jaturong_yai@hotmail.com; 4 Research Center of Microbial Diversity and Sustainable Utilization, Chiang Mai University, Chiang Mai 50200, Thailand; 5 Academy of Science, The Royal Society of Thailand, Bangkok 10200, Thailand

**Keywords:** biofermentation, thermophilic fungus, fungal enzymes, phytase, phosphorus, biomass waste

## Abstract

Agricultural wastes are lignocellulosic biomasses that contain high mineral and nutrient contents. This waste can be used as a raw material in industrial enzyme production by microbial fermentation. Phytase is an important enzyme used in animal feed to enhance the amount of phosphorus available for the growth and overall health improvement of monogastric animals. Fungi offer high potential as an effective source in the production of various extracellular enzymes. In this study, the production of lignocellulolytic enzymes (endoglucanase and xylanase) and phytase by a thermophilic fungus, namely *Thermoascus aurantiacus* strain SL16W, was evaluated using sixteen different Thai agricultural forms of waste under conditions of high temperature (45 °C). Semi-solid state fermentation was used in the production experiments. The results of this study reveal that the highest phytase activity (58.6 U/g substrate) was found in rice bran, whereas the highest degrees of activity of endoglucanase and xylanase were observed in wheat bran and red tea leaves at 19 and 162 U/g substrate, respectively. Consequently, the optimal conditions for phytase production of this fungus using rice bran were investigated. The results indicate that the highest phytase yield (58.6 to 84.1 U/g substrate) was observed in rice bran containing 0.5% ammonium sulfate as a nitrogen source with 10 discs of inoculum size at a cultivation period of 9 days at 45 °C and moisture content of 95%. Notably, the phytase yield increased by 1.71-fold, while endoglucanase and xylanase were also increased by 1.69- and 1.12-fold, respectively. Furthermore, the crude enzyme obtained from the optimal condition was extracted. The crude enzyme extract was then separately added to red tea leaves, rice straw, corncobs, palm residue, and peanut husks. Subsequently, total reducing sugar and phosphorus contents were determined. The results indicate that the highest level of reducing sugar (122.6 mg/L) and phosphorus content (452.6 mg/L) (*p* < 0.05) were obtained in palm residue at 36 and 48 h, respectively, after the addition of the crude enzyme extract. This study has provided valuable information on a potentially eco-friendly way to valorize agricultural waste into value-added products as industrial enzymes.

## 1. Introduction

In Thailand, the agricultural industry contributes significantly to the nation’s economic standing. The main agricultural products in Thailand include rice, sugarcane, cassava, corn, rubber, and palm oil, while there are a number of other such agricultural products. Consequently, over 80 million tons of agricultural waste are generated every year. A good deal of the agricultural waste could be used in animal feed, transformed into fuel or chemicals, or converted into functional bioethanol products [1]. This could then reduce the amount of waste that would need to be disposed of by burning, which is a leading cause of air pollution. In terms of its availability, and the fact that agricultural waste mainly comprises cellulose, hemicellulose, lignin, and other polysaccharides, it could also be applied as a raw material in the production of other high value-added compounds, particularly as an enzyme in the production in animal feeds. In animal feed production, lignocellulose has a negative correlation as a supplement in feed stuff, especially in the feed of certain monogastric animals like fish, poultry, and pigs. This is due to the fact that the digestive systems of these animals cannot produce the enzymes needed to digest lignocellulose. However, lignocellulose can be degraded by a variety of microorganisms, which can produce various cellulolytic enzymes, e.g., cellulase, xylanase [2], and lignin degradation enzymes (laccase, lignin peroxidase and manganese peroxidase) [3]. These enzymes have been used to treat animal feed as a way of increasing digestibility [4,5]. Additionally, a lack of phosphorus is considered a major problem in the monogastric animal feed industry because phosphorus is an important component of the metabolic process. It also plays a key role in the development of bones and the overall health of farm animals. Importantly, it is one of the major problems in the monogastric animal feed industry. Generally, feedstuffs that are based on plants, including cereal grains and their by-products, contain about 60–90% of their total phosphorus content in the form of phytates. These phytates are present in different amounts depending upon the raw plant material [6]. The storage form of phosphorus is antinutritional due to its ability to chelate various essential divalent metal ions, along with its ability to block or slow down absorption and utilization [7,8]. The insoluble forms of phytate phosphorus also act as antinutritional agents because of their ability to chelate certain essential minerals such as calcium, magnesium, iron, and zinc. Moreover, they can inhibit the action of active enzymes like phosphatase, amylase, and cellulase, in turn decreasing the digestibility of proteins, starches, and lipids [9]. These factors have led to a nutritional problem in monogastric animals. To address this concern, inorganic phosphate would need to be added to the feed of these animals. However, phosphorus that is excreted in feces has resulted in a significant amount of environmental pollution. Thus, to increase plant phosphorus utilization and to avoid the undesirable effects of phytic acid in animal nutrition, microbial phytase has been used for its beneficial effect on growth performance, along with its potential to reduce the cost of feed production. Phytase (myo-inositol hexakisphosphate phosphohydrolase) is an enzyme that can cleave phosphomonoester bonds in phytic acid and subsequently generate many of the various lower phosphate ester molecules of myo-inositol and ortho-phosphates [10,11]. This enzyme was used for animal feed and has been commercialized in recent years [12,13]. Additionally, it has been reported that various forms of feedstuff used in Belgian feed-mills, such as rye, triticale, wheat, barley, and wheat by–products, qualify as enriched sources of phytase [14]. Similarly, water hyacinth, one of the world’s worst aquatic weeds, was found to be a good source for phytase production by *Pholiota adiposa* SDBR–CMU–R32 [15]. Thus, agricultural wastes are of particular interest as substrates in fermentation because they are known to contain lignocellulose which can serve as a carbon source. Furthermore, they have also been recognized as sources of both reducing sugar and nitrogen that are required for the growth of fungal biomass. Moreover, these substrates comprise phytate molecules that are known to be inducers of phytase production.

Various microorganisms have been reported for their ability to produce cellulolytic enzymes and phytase, such as bacteria (*Actinobacillus* sp., *Bacillus pumilus*, *B. vallimortis*, and *Escherichia coli*) and fungi (*Aspergillus niger*, *A. fumigatus*, *Myceliophora thermophile*, and *Thermomyces lanuginosus*) [16]. Notably, thermophilic fungi have been of significant interest as a source of microbial enzymes because they can produce enzymes at high temperatures. Different strains of thermophilic fungus, such as *Thermoascus aurantiacus*, have been reported to be able to produce a range of enzymes such as amylases, cellulases, pectinases, and xylanases. This would indicate a high potential for effective enzyme production at high temperatures [17,18]. *Thermoascus aurantiacus* SL16W, a thermophilic fungal strain that was isolated from soil samples under a layer of decaying tree fiber, has been reported in published literature as displaying a high potential for xylanase production. Additionally, this thermophilic fungal strain has been found to be harmless in experiments that involved animal testing such as those involving male albino rats and Thai native chickens [19,20,21]. However, there have been no published reports on the production of endoglucanase and phytase from this fungal strain. Enzyme production under solid state fermentation (SSF) conditions has been reported in published studies for the last several years [22]. The conditions for fermentation generally employ biomass as both a carbon and energy source. SSF offers potential advantages over submerged fermentation (SMF) because of its low energy consumption, simple process, superior enzyme productivity, the fact that it is less susceptible to bacterial contamination, the way in which it is associated with low capital investment, and the fact that it is easy to use in terms of product recovery [23,24]. Although fungi are able to grow in low water activity environments, fungal growth inhibition has been observed under conditions of SSF at high temperatures. Both the nature and moisture of the agricultural waste that is used for SSF culture are highly critical factors [25]. Importantly, this can become a problem for enzyme production under conditions of high temperatures. On the other hand, semi-solid state fermentation (SSSF) is a type of SSF that is associated with an increase in water activity in this type of culture and provides nutrient availability for fungal growth and the control of fermentation [26]. Previous studies have reported that the enzyme contents produced under SSSF conditions were higher than those of SMF and SSF [27,28]. Accordingly, SSSF can potentially be used through an alternative set of conditions to address some of the drawbacks presented by SSF at high temperatures.

Therefore, the need to identify new microbial sources that can produce phytase efficiently at high temperatures is important for the production of animal feed with high phosphorus content in the industry. This study aimed to investigate the production of phytase and the amount of phosphorus content that is released from agricultural waste in Thailand by *T. aurantiacus* SL16W under conditions of semi-solid state fermentation. In this study, the phosphorus content will also be determined and recorded. Moreover, endoglucanase and xylanase activities and total sugar content will also be investigated.

## 2. Materials and Methods

### 2.1. Microorganisms and Inoculum Preparation

*Thermoascus aurantiacus* SL16W was kept at the Sustainable Development of Biological Resources Laboratory (SDBR, Chiang Mai, Thailand). The fungus was cultured and maintained on potato dextrose agar (PDA; Himedia, India). It was maintained by periodic transfer and stored at 4 °C. The fungus was grown on PDA for 4 days at 45 °C and then used as an inoculum for enzyme production.

### 2.2. Lignocellulosic Wastes

A total of 16 different lignocellulosic wastes, including mango peel, green tea leaf, corncobs, red tea leaf, rice straw, pineapple peel, rice bran, bamboo husk, soybean husk, wheat bran, pineapple leaf, dragon fruit peel, peanut husk, palm residue, bamboo pulp, and coffee cherry peel were used in this study. All substrates were dried at 60 °C for 3 days. They were then cut and ground with a grinder (0.25–0.50 cm) before use.

### 2.3. Effect of Various Substrates on Phytase, Endoglucanase, and Xylanase Production by Semi-Solid State Fermentation (SSSF) Procedure

SSSF was carried out in 250 mL Erlenmeyer flasks containing 5 g of dried substrate (0.25–0.50 cm) and 50 mL of distilled water (1:10) in each flask. The substrate was sterilized at 110 °C for 20 min. After the flasks were allowed to cool down, the substrate was inoculated with 10 discs (8 mm diameter) of 4-day-old inoculum. The flasks were then incubated at 45 °C for 7 days. The enzyme was extracted and assessed for phytase, endoglucanase, and xylanase activities. All experiments were conducted in triplicate. Consequently, a suitable substrate was selected for further experimentation. SSSF was carried out in order to study the effect of various parameters required for the optimum production of phytase, endoglucanase, and xylanase by *T. auratiacus* SL16W from agricultural waste.

### 2.4. Extraction and Determination of Enzyme Activity

#### 2.4.1. Enzyme Extraction

After SSSF, a total of 50 mL of 50 mM sodium acetate buffer (pH 5.0) were added to each flask. The flasks were shaken on a rotary shaker at 150 rpm for 1 h at room temperature. The suspension was then filtrated and the final volume was adjusted to 100 mL. The filtrated solution was centrifuged at 6000 rpm at 4 °C for 10 min and the clear supernatant was used as a crude enzyme. Subsequently, the solution was stored at 4 °C for further preparation of the enzyme assay.

#### 2.4.2. Phytase Assay

Phytase activity was determined by measuring the amount of inorganic phosphorus released from the sodium phytate solution using the method described by JECFA [29] with slightly modifications. Sodium phytate (0.0051 mol/L) substrate was dissolved in 50 mM acetate buffer pH 5.0. The substrate was then mixed with a crude enzyme and incubated in a water bath at 50 °C for 30 min. After incubation, the reaction was stopped by adding Taussley–Schor’s reagent into the tubes. After allowing the solution to cool down to room temperature, levels of absorbance were measured spectrophotometrically at 660 nm. The blank was determined to be the substrate that had been inoculated with sterile deionized (DI) water. The data obtained were used to calculate the activity unit of phytase (unit/gram substrate; U/g substrate). One phytase unit was defined as the amount of enzyme releasing 1 µmol of inorganic phosphorus per minute from 0.0051 mol/L sodium phytate under the test conditions.

#### 2.4.3. Endoglucanase Assay

Endoglucanase activity of carboxymethyl cellulose (CMCase) was measured according to the method described previously by Ghose [30] using a reaction mixture containing 180 µL of 1% carboxymethyl cellulose (CMC) in 50 mM acetate buffer (pH 5.0) and 20 µL of enzyme solution. The reaction mixture was incubated at 50 °C for 30 min and the amount of reducing sugar produced was determined using the 3,5-dinitrosalicylic acid (DNS) assay. One unit of endoglucanase activity was defined as the amount of enzyme that released 1 µmol of reducing sugar per minute under standard assay conditions.

#### 2.4.4. Xylanase Assay

Xylanase was assayed according to the method of Bailey et al. [31] by measuring the amount of reducing sugar (xylose equivalent) liberated from xylan using DNS assay. The reaction mixture containing 20 µL enzyme extract and 180 µL xylan solution (1% *w/v* in 50 mM acetate buffer, pH 5.0) was incubated at 50 °C. After 30 min, the reaction was stopped by adding 300 µL of DNS reagent and the amount of reducing sugar released in the reaction was estimated by measuring the absorbance at 550 nm. One unit of xylanase activity was defined as the amount of enzyme required to release 1 µmol xylose from xylan per minute under standard assay conditions.

### 2.5. One Factor at a Time Optimization (OFAT) for Phytase, Endoglucanase and Xylanase Production

The initial batch studies were carried out by applying the OFAT method and by varying only a single factor while keeping the remaining factors constant.

#### 2.5.1. Effect of Different Sugars

Five grams of rice bran (RB) and 50 mL of distilled water were prepared in different flasks and each flask was supplemented with a different sugar at 0.01% (*w/v*). The various sugars used were lactose, ribose, xylose, galactose, and arabinose. The substrate was sterilized at 110 °C for 20 min. Each media flask was then inoculated with 10 discs (8 mm diameter) of 4-day-old mycelium and incubated at 45 °C for 7 days. After 7 days, the enzyme was extracted and estimated for phytase, endoglucanase, and xylanase activities.

#### 2.5.2. Effect of Different Nitrogen Sources

To determine the effects of different nitrogen sources on phytase, endoglucanase, and xylanase production, 5 g of rice bran (RB) and 50 mL of distilled water were supplemented with 0.5% (*w/v*) of different nitrogen sources such as urea, ammonium chloride (NH_4_Cl), ammonium nitrate (NH_4_NO_3_), ammonium sulfate (NH_4_(SO_4_)_2_), and ammonium acetate (NH_4_CH_3_CO_2_). The fermentation substrate was then inoculated with 10 mycelium discs (8 mm diameter) of 4-day-old mycelium and incubated at 45 °C for 7 days. The fermented supernatant was then extracted and collected and the level of activity of the three enzymes was estimated.

#### 2.5.3. Effect of Amount of Fungal Inoculum

Five grams of rice bran (RB) and 50 mL of distilled water were prepared in different flasks and each flask was inoculated with a different amount of fungal inoculum, which varied over a range of 5–50 discs (8 mm diameter) of 4-day-old mycelium. Flasks were then incubated at 45 °C for 7 days while all other factors were kept constant. The fermented solution was extracted and collected, and the level of activity of the three enzymes was estimated.

#### 2.5.4. Effect of Fermentation Period

The fermentation period was studied by varying the incubation time at 3, 6, 9, 12, and 15 days. Five grams of rice bran and 50 mL of distilled water were prepared in different flasks and each flask was then inoculated with 10 discs (8 mm diameter) of 4-day-old mycelium. The specimens were then incubated at 45 °C for 3 to 15 days while all other factors were kept constant. The fermented solution was extracted and collected, and the level of activity of the three enzymes was estimated.

#### 2.5.5. Effect of Moisture Content

The optimal level of moisture content was studied by using the volumes of 5, 10, 25, 50, and 100 mL of distilled water with 5 g of rice bran (RB). Each medium flask was then inoculated with 10 discs (8 mm diameter) of 4-day-old mycelium. The specimens were then incubated at 45 °C for 9 days while all other factors were kept constant. The fermented solution was extracted and collected, and the level of activity of the three enzymes was estimated.

### 2.6. Enzyme Fermentation

Five liters of extracted enzyme solution were obtained from the optimized conditions. One hundred milliliters of the extracted enzyme were fermented with 5 g of different agricultural substrates including red tea, corncobs, rice straw, palm residue, and peanut husks. The fermented flasks were incubated at 50 °C for 12, 24, 36, and 48 h. The fermented solution was extracted and collected, and then total reducing sugar and phosphorus contents were analyzed. The concentration values of the produced reducing sugars were estimated using DNS method with glucose as the standard [32]. Determination of the phosphorus content was calculated using the method of AOAC [33].

### 2.7. Statistical Analysis

Statistical analyses were performed using SPSS software (SPSS 18.0 for windows; SPSS Inc., Chicago, IL, USA). The significance of enzyme production with regard to the differing parameters was determined by an ANOVA procedure, while Duncan’s multiple comparison test was used to determine any significant differences (*p* < 0.05) that were identified between treatments.

## 3. Results

### 3.1. Effect of Various Substrates on Phytase, Endoglucanase and Xylanase Production under SSSF

**Figure 1:**
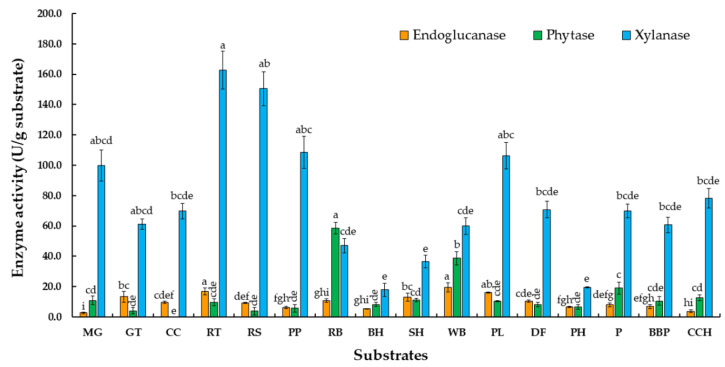
Effect of agricultural waste on phytase, endoglucanase, and xylanase production from various substrates (mango peel (MG), green tea leaf (GT), corncob (CC), red tea leaf (RT), rice straw (RS), pineapple peel (PP), rice bran (RB), bamboo husk (BH), soybean husk (SH), wheat bran (WB), pineapple leaf (PL), dragon fruit peel (DF), peanut husk (PH), palm residue (P), bamboo pulp (BBP), and coffee cherry peel (CCH)) by *Thermoascus aurantiacus* SL16W under semi-solid state fermentation after being incubated at 45 °C for 7 days. Average ± standard deviation from three replicate experiments. Different letters in the same column are considered significantly different according to Duncan’s multiple comparison test (*p* ˂ 0.05).

Plant lignocellulosic biomass has been recognized as a potential substrate for the production of enzymes. In order to determine the influence of agricultural residue on phytase, endoglucanase, and xylanase production, carbon sources present in the production medium were replaced with a range of various agricultural wastes at 10% (*w/v*) concentrations. The results in Figure 1 indicate that phytase displayed maximum activity (58.6 U/g substrate) in rice bran, followed by wheat bran and palm residue. The highest content of endoglucanase was 19 U/g substrate when wheat bran was used as a substrate. Xylanase activity revealed maximum activity (162 U/g substrate) in red tea leaf.

### 3.2. One Factor at a Time Optimization (OFAT) for Phytase, Endoglucanase and Xylanase Production

This experiment was performed to examine the effect of different sugars, nitrogen sources, inoculum sizes, cultivation periods, and moisture contents for phytase, endoglucanase, and xylanase production from *Thermoascus aurantiacus* SL16W under SSSF conditions.

#### 3.2.1. Effect of Different Sugars and Nitrogen Sources

**Figure 2:**
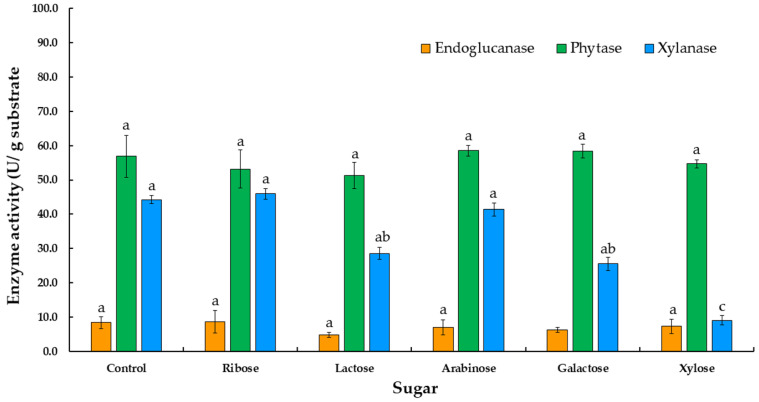
Phytase, endoglucanase, and xylanase production by *Thermoascus aurantiacus* SL16W with different sugars under semi-solid state fermentation. Average ± standard deviation from three replicate experiments. Different letters in the same column are considered significantly different according to Duncan’s multiple comparison test (*p* ˂ 0.05).

The production of phytase, endoglucanase, and xylanase in the medium of rice bran under SSSF conditions supplemented with different sugars was assessed. The results showed that there were no significant differences among the sugars in terms of phytase, endoglucanase, and xylanase production (Figure 2), including lactose, ribose, xylose, galactose, and arabinose. Consequently, sugars did not affect enzyme production.

**Figure 3:**
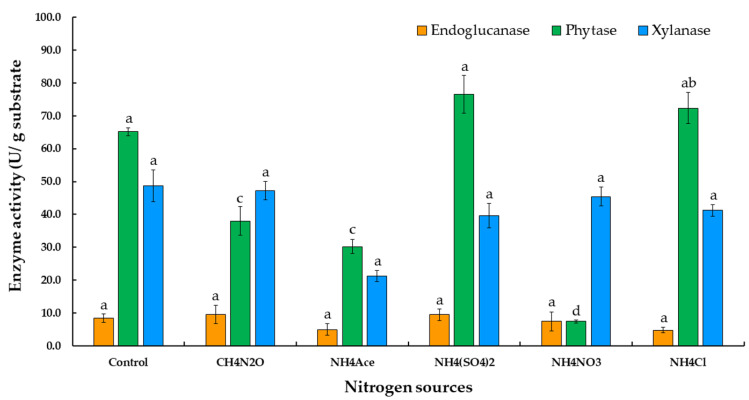
Phytase, endoglucanase, and xylanase production by *Thermoascus aurantiacus* SL16W with various nitrogen sources under semi-solid state fermentation. Average ± standard deviation from three replicate experiments. Different letters in the same column are considered significantly different according to Duncan’s multiple comparison test (*p* ˂ 0.05).

In addition, when comparing the nitrogen sources for phytase, endoglucanase, and xylanase production of urea, ammonium acetate, ammonium sulfate, ammonium nitrate, and ammonium chloride, the results presented in Figure 3 indicate that ammonium sulfate presented the best substrate for phytase and endoglucanase production in this experiment. Therefore, ammonium sulfate was selected as a nitrogen source for the production of the three enzymes in the further experiments.

#### 3.2.2. Effect of Amount of Fungal Inoculum

**Figure 4:**
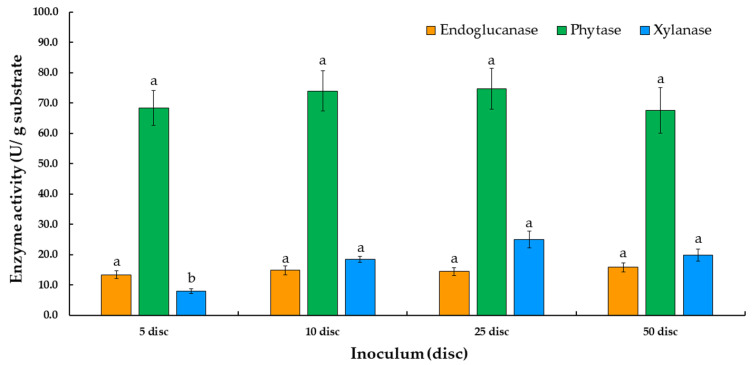
Phytase, endoglucanase, and xylanase production by *Thermoascus aurantiacus* SL16W at various inoculum sizes under semi-solid state fermentation. Average ± standard deviation from three replicate experiments. Different letters in the same column are considered significantly different according to Duncan’s multiple comparison test (*p* ˂ 0.05).

The results of the amount of fungal inoculum on the production of phytase, endoglucanase, and xylanase are presented in Figure 4. A fungal inoculum of 10 discs was found to be optimal for the exhibition of the highest degree of phytase activity at 73.9 U/g substrate by *T. aurantiacus* SL16W (*p* < 0.05). Thus, this inoculum size was selected for use in subsequent experiments.

#### 3.2.3. Effect of Fermentation Period

**Figure 5:**
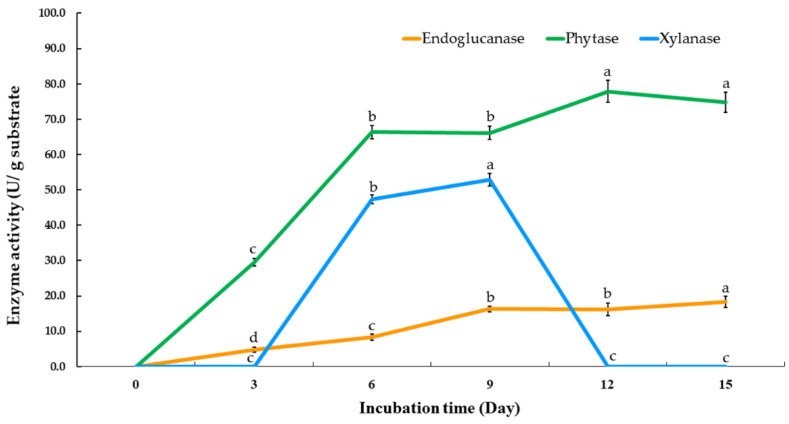
Time course of phytase, endoglucanase, and xylanase production by *Thermoascus aurantiacus* SL16W under semi-solid state fermentation. Different letters in the same column are considered significantly different according to Duncan’s multiple comparison test (*p* ˂ 0.05).

The highest degree of phytase activity of 77.8 U/g substrate was detected at 12 days of fermentation (Figure 5). This result clearly indicates that xylanase activity sharply decreased after 9 days, and endoglucanase activity slightly increased from 3 to 12 days. The optimal incubation time was found to be 9 days and will be applied in further experiments.

#### 3.2.4. Effect of Moisture Content

**Figure 6:**
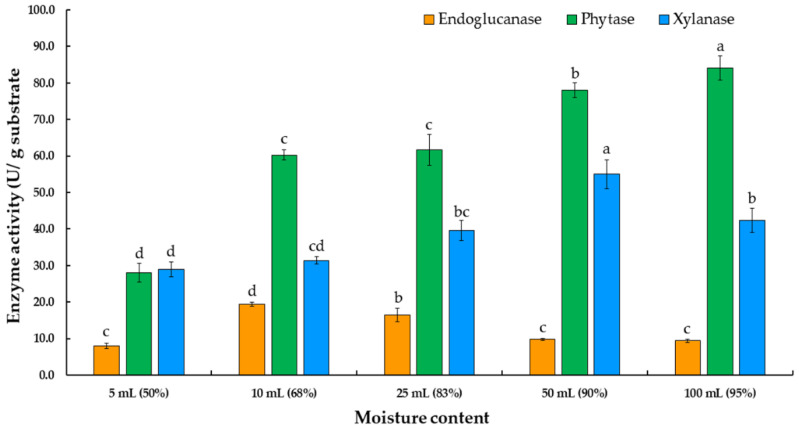
Phytase, endoglucanase, and xylanase production by *Thermoascus aurantiacus* SL16W at various moisture contents under semi-solid state fermentation. Average ± standard deviation from three replicate experiments. Different letters in the same column are considered significantly different according to Duncan’s multiple comparison test (*p* ˂ 0.05).

Suitable moisture content for phytase, endoglucanase, and xylanase production was also checked by changing the water volume in the 250 mL Erlenmeyer flasks from 5 to 100 mL. Of all these, the tested moisture content of 100 mL with 5 g of the substrate resulted in the optimum degree of phytase production (84.1 U/g substrate), as is shown in Figure 6.

### 3.3. Enzyme Fermentation

**Figure 7:**
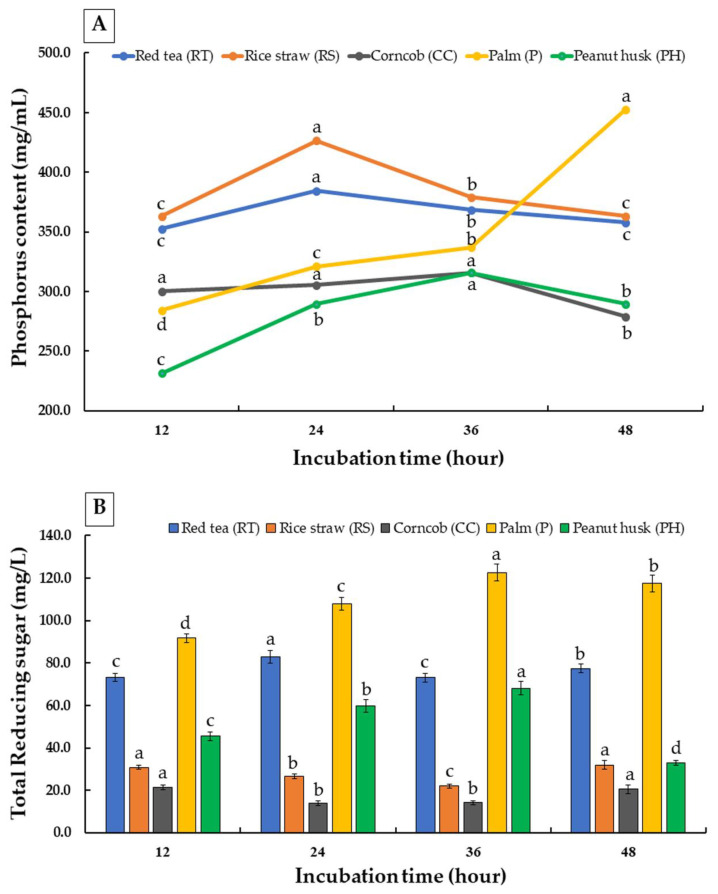
Phosphorus (**A**) and total reducing sugar (**B**) content of crude enzyme extract obtained from *Thermoascus aurantiacus* SL16W using different substrates: red tea, rice straw, corncob, palm residue, and peanut husk. Different letters in the same column are considered significantly different according to Duncan’s multiple comparison test (*p* ˂ 0.05).

The different substrates, such as red tea, rice straw, corncobs, palm residue, and peanut husks, were incubated with the crude enzyme extract from *T. aurantiacus* SL16W at 50 °C for 48 h. The supernatant of rice straw showed the highest phosphorus content at 426 mg/L for 24 h, while palm residue possessed the highest phosphorus content at 452.6 mg/L after 48 h of incubation (Figure 7A). Moreover, the total amount of reducing sugar in the palm residue was higher than in the other forms of biomass (Figure 7B). Rice straw and palm residue have been suggested as components of animal feed. 

## 4. Discussion

Agricultural residues offer potential as a low-cost feedstock that can provide natural carbon sources for enzyme production. Notably, SSF conditions are commonly used in enzyme production, particularly cellulase and xylanase [22]. Furthermore, commercially available exogenous phytases are commonly derived from microorganisms using SSF, SSSF, and SMF [34]. The major advantage of fungal SSF is that the fungi can grow on complex natural substrates. However, the disadvantages of SSF include that it is associated with slow nutrient activity and low biomass growth. This tends to occur because of a lack of free water content along with an accumulation of heat and a loss of moisture during the fermentation process [35,36]. Remarkably, the production of enzymes takes place under conditions of high temperature. Accordingly, SSSF can be employed to minimize the problems associated with SSF. A previous study conducted by Gupta and Jana [28] revealed that the solid contents of agriculture residue in a fermentation medium (2‒20% *w/v*) can affect the production of laccase. In terms of the wheat straw content (8% *w/v*) in SSSF, the fungal strain *Ganoderma lucidum* could be applied as both a soluble and insoluble nutrient indicating the greatest degrees of growth and laccase activity. The enzyme activity in SSSF was higher by ~3.5-fold than that of SMF and ~2.5-fold higher than that of SSF. These outcomes may have been due to the fact that SMF fungus can exploit only soluble nutrients and was incapable of accessing insoluble nutrients in the media due to a lack of contact with those solids. However, the fungus utilized mainly insoluble nutrients and could grow on solid surfaces with dense and thin hyphae under conditions producing large amounts of solid contents (SSF).

This study evaluated the production of endoglucanase, xylanase, and phytase by *T. aurantiacus* strain SL16W using various forms of agricultural waste (10% *w/v*) under SSSF conditions of high temperature (45 °C). The phytase production potential of *T. aurantiacus* SL16W on agricultural waste revealed a preference of the test strain for rice bran, resulting in the highest degree of enzyme activity (58.6 U/g substrate), so rice bran was selected as a substrate to be used in further experiments. These results are similar to those of previous studies [37,38,39] which suggested that rice bran encouraged phytase production, while wheat bran was also found to act as a potentially good substrate [40,41,42]. However, this result did not correlate with the finding of a previous report conducted by Gaind and Singh [11] which suggested that different varieties of cereal bran (wheat and rice) could be effective substrates for low phytase production of *Aspergillus niger* ITCC6720. Additionally, Pires et al. [43] reported that the fungus *Acremonim zeae* exhibited the highest degree of phytase production in a medium containing cornmeal, while the yeast *Kluyveromyces marxianus* produced 10-fold more phytase than *A. zeae* when cultivated on rice bran. The study conducted by Bujna et al. [6] determined that a thermophilic fungus, *Th. lanuginosus* IMI 096218, could produce phytase by using rice flour as a carbon source. Furthermore, the study conducted by Jatuwong et al. [15] revealed that the highest level of phytase activity (53.66 U/g substrate) was observed with *Pholiota adiposa* in water hyacinth containing 85% moisture content supplemented with a basal medium at a pH value of 6.5 after being incubated at 30 °C for 7 days. Therefore, the activity of phytase obtained from a microbial species was found to be directly dependent upon the type of substrate and fungal species, as well as the actual fungal strain. Additionally, the highest degrees of activity of endoglucanase and xylanase in this study were observed in wheat bran (19 U/g substrate) and red tea leaves (162 U/g substrate), respectively. In the study conducted by Brijwani et al. [44], the cellulase activity of *Trichoderma reesei* and *A. oryzae* upon solid state fermentation using soybean hulls and wheat bran at a 4:1 ratio was 10.7 U/g substrate after optimization. However, xylanase activity in this study resulted in slightly lower levels than were found in a previous study conducted by Chawachart et al. [21] who had reported on *T. aurantiacus* SL16W using corncob as a substrate under conditions of solid state fermentation (987 U/g substrate). Moreover, there have been a number of reports on xylanase production by other microorganisms; for example, *Tr. reesei* Rut C–30 has been reported for its significant capability of xylanase production (248 U/mL) [45]. Thermo fungus *T. aurantiacus* RCKK produced a high level of xylanase of 6245 U/g substrate under conditions of solid state fermentation [46]. Notably, thermophilic fungi are known to be xylanase-producing microorganisms, including strains of *Thermomyces*, and they have been reported to be among the best xylanase producers in nature [47]. 

Moisture content would need to be optimized for maximum fungal growth and the production of enzymes. Additionally, the evaluation of some parameters, such as the addition of sugars and nitrogen sources, the amount of inoculum present, and the length of the fermentation period, support the maximum amount of enzyme production under SSSF conditions. A previous study has revealed that the supplementation of a carbon source, along with moisture content, inoculum level, inoculum age, and cultivation period, as well as incubation temperature and pH, could enhance phytase activity [11]. The supplementation of different sugars used in this study, including lactose, ribose, xylose, galactose, and arabinose, had no significant effect on phytase, endoglucanase, and xylanase production. On the other hand, the addition of various nitrogen sources for phytase, endoglucanase, and xylanase production, such as urea, ammonium acetate, ammonium sulfate, ammonium nitrate, and ammonium chloride, was also investigated. Ammonium sulfate was found to be the best nitrogen source for phytase, endoglucanase, and xylanase production in this experiment. Therefore, ammonium sulfate has been selected as a nitrogen source for the production of the three enzymes in a subsequent study. Previous publications reported that ammonium sulfate is a nitrogen source that is necessary for microbial growth. Kanpiengjai et al. [38] found that when ammonium sulfate (2.64% *w/v*) was added to the basal medium, phytase production of *Anoxybacillus* sp. MHW14 increased to 0.20 U/mL. The optimization of phytase production was achieved with the yeast *Kodamaea ohmeri* BG3 in a cost-effective oat medium containing 1.0% oats, 2.3% ammonium sulfate, 2.0% glucose, and 2.0% NaCl, which was found to be the optimal medium [48]. Similarly, fungal phytase production indicated that ammonium sulfate was an effective nitrogen supplement. The study of Tian and Yuan [49] reported that adding 4% ammonium sulfate to the medium could result in a maximum amount of phytase at 12.93 ± 0.47 U/g dry substrate. According to the statistical results of this study, the inoculum size of 10 discs exhibited the highest degree of phytase activity, while 5 and 50 discs of fungal inoculum revealed no statistically significant differences between phytase and endoglucanase activities. Moreover, although there was no statistically significant effect on enzyme activity using 5 and 50 discs of fungal inoculum (*p* > 0.05), there was a sharp increase in phytase, endoglucanase, and xylanase production by *T. aurantiacus* SL16W between 3 and 15 days of fermentation. Although the highest degree of phytase activity was found after 12 days of fermentation, a fermentation period of 9 days will be applied in an experiment involving enzyme fermentation. This is due to the fact that xylanase was not found after 9 days of fermentation.

Total reducing sugar and phosphorus contents were investigated using the fermentation medium of the extracted enzyme that was derived from the optimized medium. This was achieved along with the specified optimized conditions (rice bran containing 0.5% ammonium sulfate as a nitrogen source with 10 discs of inoculum in a cultivation period of 9 days at 45 °C and moisture content of 95%) for fermentation with 5 g of different agricultural substrates including red tea, corncobs, rice straw, palm residue, and peanut husks. The specimens were then incubated at 50 °C for 12, 24, 36, and 48 h. The highest amount of reducing sugar (122.6 mg/L) and phosphorus content (452.6 mg/L) (*p* < 0.05) were found in the supernatant of palm residue after periods of incubation of 36 and 48 h, respectively. These results indicate the beneficial effect of phytase extract derived from *T. aurantiacus* SL16W that had been cultivated in rice bran on the biological availability of dietary phosphorus. In a previous study, the use of at least 1000 U/kg of fungal phytase included in corn/soybean mealbased diets of pigs increased the degree of phosphorus retention from 52 to 64% [50]. The studies of Simon et al. [51] and Kornegay et al. [52] also found that phosphorus retention by broilers was improved from 50–60% after being fed diets supplemented with fungal phytase. Therefore, when feedstuffs were incubated with phytase, a certain amount of inorganic phosphorus could be removed from the diet. Moreover, the typical amount of phosphorus that was removed for 500 U of phytase/kg diet could vary from 0.06% to 0.01% for broilers, turkeys, and swine, all of which resulted in the degree of phosphorus excretion being reduced in the range of 15–30% [53]. Agricultural biomass is not recommended to be used as direct fertilizer because the available literature has proven that the application of undecomposed waste or nonstabilized compost to agricultural land may lead to the immobilization of plant nutrients and could cause phytotoxicity [54]. Moreover, the presence of monosaccharide/sugars (e.g., xylose, arabinose, galactose, and mannose), which were derived from the hydrolysis of hemicellulose, could be assembled into ethanol, CO_2_, and H_2_O and then be broken down into pentoses and CO_2_ [55]. Thus, other safe forms of disposal and environmentally friendly methods of management of agricultural biomass, such as those involving enzyme production and feedstock, are presently being investigated. Based on these results, phosphorus could be produced from fungal enzyme extracts using low-cost agricultural biomass with a strong potential for applications in hydrolyzing the phytates present in the feedstuffs of monogastric animal feeds. Feed enzymes may be used in animal diet formulations, which can then be added to degrade specific feed components [56]. The study of Shahryari et al. [57] revealed that a high degree of fungal phytase activity on wheat straw resulted a decrease in phytic acid content by 57.4% when compared to the untreated sample. Thus, feed enzymes can increase the digestibility of nutrients and result in higher feed utilization by animals [58]. Importantly, these results indicate that crude enzymes were obtained from *T. aurantiacus*, which has been classified as a fungus of biosafety level 1 [59]. Furthermore, crude enzymes were found to be harmless to phytase. These outcomes were supported by the findings of a research study conducted by Kongbantad et al. [19] who reported that the crude enzyme derived from *T. aurantiacus* SL16W showed no toxic effects on the hematological system, as well as on the function of the liver and kidneys of albino rats. However, enzyme stability and toxicity assays are also necessary in the laboratory, while clinical tests are needed in future studies to fully understand the profile of this fungus. In addition, there is a need to further improve upon the amounts of enzymes used in genetic engineering procedures, while the use of modern techniques at the downstream processing stage could be employed for the improved activity of those enzymes.

## 5. Conclusions

The present study has shown that *T. aurantiacus* SL16W could produce phytase, as well as endoglucanase and xylanase, on different forms of agricultural waste in addition to liquid media without the supplementation of a carbon source at high temperatures under SSSF. Furthermore, *T. aurantiacus* SL16W was found to be an excellent fungus for the production of phytase enzymes, of which rice bran was determined to be an efficient substrate for phytase production in this experiment (84.1 U/g substrate). It is clear that the crude enzymes obtained from *T. aurantiacus* SL16W, when applied to agricultural biomass, especially rice straw and palm residue, were critical in converting phytic acid to available phosphorus. This could account for the absence of phosphorus that is needed for growth and would then reduce the need for the supplementation of inorganic phosphorus in animal feeds. Therefore, the exploitation of biomass offers great potential in terms of reducing production costs and for increasing the use of enzymes in monogastric animal feed. Importantly, SSSF can encourage the production of enzymes using lignocellulosic materials as substrates at high temperatures. Furthermore, a subsequent study will be needed on the fermentation production of phytase in a laboratory-scale fermenter in order to scale up phytase production and examine the use of a phytase crude enzyme in poultry feed production.

## Data Availability

Not applicable.
